# PPAR*δ*: A Potential Therapeutic Target for the Treatment of Metabolic Hypertension

**DOI:** 10.1155/2019/7809216

**Published:** 2019-04-03

**Authors:** Yanli Jiang, Qiang Li, Mengxiao Jia, Zhencheng Yan

**Affiliations:** Department of Hypertension and Endocrinology, Center for Hypertension and Metabolic Diseases, Daping Hospital, Army Medical University, Chongqing Institute of Hypertension, Chongqing 400042, China

## Abstract

High blood pressure and its associated cardiovascular diseases have been major risks for public health. Multiple metabolic risk factors can cause the vascular dysfunction and vascular lesion, and the hypertension due to metabolic disturbances was defined as metabolic hypertension. The members of a subfamily of the nuclear receptors, peroxisome proliferator-activated receptors (PPARs), were found to be key regulators of metabolism and vascular function. We provide up-to-date knowledge on the role of subtype PPAR*δ* in the regulation of metabolism and vascular function and the effect of its intervention on the metabolic hypertension management. We hope to give some insights into the development of more effective treatments of metabolic hypertension and its main complications.

## 1. Introduction

Hypertension has been a major risk for public health, which is responsible for at least 9.4 million deaths worldwide every year [1]. Although multifactors, including genetic and secondary etiology, play important roles in the development of hypertension, roughly 60% of the risk factors for hypertension are associated with metabolic disorders [2]. Furthermore, multiple metabolic risk factors can cause the vascular dysfunction and vascular lesion. Metabolic abnormalities can directly lead to high blood pressure, and the hypertension due to metabolic disturbances was defined as metabolic hypertension [3]. According to the types of metabolic abnormalities, metabolic hypertension mainly includes obesity-related hypertension, diabetic hypertension, familial dyslipidemia-associated hypertension, metabolic syndrome, hypertension with hyperhomocysteinemia, hypertension with hyperuricemia, and salt-sensitive hypertension [3, 4].

The association between metabolic risk factors and hypertension raises important attention for the underlying pathological processes, especially for focusing intervention target. The members of a subfamily of the nuclear receptors, peroxisome proliferator-activated receptors (PPARs), were found to be key regulators of metabolism and vascular function. Three subtypes including PPAR*α*, *γ*, and *β*/*δ* have been described. PPAR*δ* is ubiquitously expressed, which can be activated by long-chain fatty acids and thus acted as fatty acid sensors regulating a variety of genes implicated in lipid metabolism [5]. Next to fatty acids, it has been shown that prostacyclin [6] and retinoic acid [7] efficiently activate PPAR*δ*-mediated transcription and thus participated in the blastocyst implantation and enhanced the transcriptional activity of PPAR*δ*. Synthetic compounds of PPAR*δ* agonists have been developed, GW501516 and GW0742, with a 1000-fold selectivity over the other PPAR isotypes [8]. When activated, PPAR*δ* promotes fatty acid oxidation, thermogenesis, insulin sensitivity, high density lipoprotein cholesterol (HDLc) levels in plasma, and overall energy expenditure [9]. The reports about the influence of PPAR*δ* on vascular diseases and thereby blood pressure regulation in recent years have attracted much attention.

This review suggests that PPAR*δ* might be a potential important regulator in the pathogenesis of metabolic hypertension. We provide up-to-date knowledge on the role of PPAR*δ* in the regulation of metabolism and vascular function, and the effect of PPAR*δ* intervention on metabolic hypertension management.

## 2. The Effects of PPAR**δ** on Glucose and Lipid Metabolism Regulation

Among three subtypes of PPARs, PPAR*α* and PPAR*γ* have been widely applied in clinic because the related drugs are already available for treating metabolic disease including type II diabetes and dyslipidemia (such as PPAR*α* agonist fibrates and PPAR*γ* agonist thiazolidinediones) [10]. However, the physiology characteristics of PPAR*δ* are less investigated. In recent years, with the discovery of high potent and selective agonists [11, 12], the fact that PPAR*δ* is playing an important role in glucose and lipid metabolism, inflammatory response, cell survival, wound healing, embryo transplantation, and the development of central nervous system has been confirmed.

PPAR*δ* is expressed almost ubiquitously, with the highest level of expression found in colon, small intestine, liver, and keratinocyte. PPAR*δ* is a general regulator of fatty acid oxidation in many tissues. The involvement of PPAR*δ* in the regulation of lipid metabolism has been well established based on knockout and overexpression studies in transgenic mice [13]. Activation of PPAR*δ* in adipose tissue could improve lipid profiles and reduce adiposity because of the expression of genes involved in fatty acid oxidation and energy dissipation [14]. Overexpression of a constitutive active PPAR*δ* in white adipose tissue reduced adiposity, most probably leading to the increased level of fatty acid oxidation and therefore inducing fatty acid metabolism, mitochondrial respiration, and programming of the muscle fiber type by PPAR*δ* metabolic pathways. All the above metabolic effects improved by activation of PPAR*δ* play a crucial role in the prevention of metabolic hypertension.

## 3. The Effects of PPAR**δ** on Vascular Pathophysiological Mechanism

Vascular endothelial cells play important roles in regulating vascular permeability and maintaining the normal blood dynamics. PPAR*δ* is expressed in both human umbilical artery endothelial cells and aorta endothelial cells. Sandra Suarez et al. found that PPAR*δ* antagonism has a protective effect on vascular permeability through reducing the expression of vascular endothelial growth factor receptor (VEGFR). The VEGFR can decrease the expression of signaling pathways downstream of receptors in ERK1/2 and Akt signaling pathways [15, 16]. In addition, PPAR*δ* also can inhibit the generation of actin cytoskeleton and thus the high permeability of blood vessel induced by VEGFR is suppressed [17]. PPAR*δ* agonist GW501516 plays a positive role in the development of angiogenesis by activating nuclear factor of activated T-cells, cytoplasmic 3 (NF-ATc3), and HIF-1*α* [18]. GW501516 also can stimulate the proliferation of endothelial cells and induce angiogenesis through the expression of VEGF or other angiogenic molecules, thereby promoting the growth of capillaries and increasing the blood flux [19]. Furthermore, it has been suggested that VEGF-C is an osmosensitive, hypertonicity-driven gene intimately involved in salt-induced hypertension, since the TonEBP–VEGF-C signaling in mononuclear phagocyte system (MPS) cells is a major determinant of extracellular volume and blood pressure homeostasis [20]. Besides, PPAR*δ* plays an instrumental role in protecting endothelial cells from oxidant stress mediated apoptosis because of the upregulation of 14-3-3-*α* protein and promotion of proliferation and angiogenesis [21]. Hence, PPAR*δ* has the ability to (i) regulate certain signaling molecules such as VEGF associated with proliferation and angiogenesis of endothelial cells and (ii) control the form and function of endothelial cells and the formation of new blood vessels. Meanwhile, with a genomic and proteomic analysis of PPAR*δ*^−^ endothelial cells from Matrigel plugs, an abundance of potential proangiogenic genes for PPAR*δ* is discovered. These candidates can increase the expression of CDKN1C (cyclin-dependent kinase inhibitor 1c), which has the ability to encode cell cycle inhibitor p57Ki^p2^ [22]. In addition, for PPAR*δ* gene knockout mice, the expression of CLIC4 (Cl^−^ intracellular channel protein-4) which performs important functions during angiogenesis is decreased, whereas the expression of CRBP1 (cellular retinol binding protein-1) is increased, which inhibits the Akt survival pathway [23–25].

Vasodilatation is closely related to endothelial cells, namely, endothelium-dependent relaxation. PPAR*δ* has been implicated in the modulation of vascular homeostasis. In the aorta, PPAR*δ* agonist of high selectivity can significantly improve the diastolic function of damaged blood vessels. This effect is established by activation of endothelial NO synthase (eNOS), rather than by induction of gene expression. With an acute transcription-independent regulation of eNOS phosphorylation, the eNOS activity and NO content are both enhanced [26]. Paeonol reversed the impaired endothelium-dependent relaxations by inhibition of ER stress and oxidative stress, thus elevating NO bioavailability via the AMPK/PPAR*δ* signaling pathway, while these beneficial effects of paeonol were diminished in PPAR*δ* knockout mouse aortas [27]. PPAR*δ* can promote proliferation, angiogenesis, and vasodilatation of vascular endothelial cell as well as reducing harmful inflammation.

The increased blood pressure is associated with the changes in the vascular structure and function. The vascular smooth muscle cells (VSMCs) directly drive the contraction of the vascular wall and hence regulate the blood pressure. The activation of PPAR*δ* had been shown to suppress both the proliferation and the inflammation of VSMCs, which thereby exerted beneficial effects in preventing vascular remodeling [28, 29]. A selective ligand for PPAR*δ*, L-165041, was shown to inhibit the proliferation and migration of VSMCs through causing the cell cycle arrest by upregulating p27^kip1^ and downregulating cyclin D1 and CDK4 [30]. Angiotensin II (Ang II) was known to have a key role in the pathogenesis of hypertension through vascular remodeling, oxidative stress, and inflammatory response [31]. PPAR*δ* activation by GW0742 was found to inhibit Ang II signaling and thereby lowering blood pressure through upregulating G-protein coupled signaling (RGS) proteins 5 (RGS5) [32]. Previous study shows that the balance between the protein kinase B (Akt) and the ERK pathways may determine the differentiated phenotype of VSMCs. Zarzuelo MJ et al. found that activation of PPAR*δ* by GW0742 may reduce the vascular remodeling through increasing Akt phosphorylation while reducing ERK1/2 phosphorylation in SHR aortas [33]. Apoptosis of VSMCs was another important factor influencing vasculature function. The oxidized low-density lipoprotein (oxLDL), which is the component of atherosclerotic lesions, has been shown to induce ROS modulated cell death in VSMCs via activation of mitogen-activated protein kinases (MAPKs) [34]. Hwang JS et al. found that PPAR*δ* could modulate oxLDL-induced apoptosis of VSMCs via a TGF-*β*/FAK signaling axis, which thus participated in the progression of atherosclerosis and restenosis [35]. Furthermore, PPAR*δ* had also been found protecting brain and renal function through regulating vascular function. Yin K-J et al. studied the potential role of vascular PPAR*δ* in ischemic brain injury, and they found that the PPAR*δ* deletion could result in the increase of cerebrovascular permeability and brain infarction in mice after middle cerebral artery occlusion [36]. Kirkby NS et al. found that renal blood flow is regulated by cyclooxygenase-2 (COX-2) activity through PPAR*δ*-mediated renal vasodilator pathway involving prostacyclin, indicating the potential of COX-2/prostacyclin/PPAR*δ* axis as a therapeutic target in renal disease [37].

## 4. Activation of PPAR**δ** Attenuates Metabolic Hypertension

### 4.1. PPAR*δ* Activation Prevents Obesity

The incidence of obesity increases dramatically in the past decades. High levels of low density lipoprotein (LDL-c) and very low density lipoprotein (VLDL), and lower level of high density lipoprotein cholesterol (HDL), are major factors in metabolic abnormalities associated with obesity and cardiovascular mortality [38]. William R. Oliver et al. found that GW501516 belonging to PPAR*δ* agonists could enhance the expression of HDL-c with 79% and decrease the expression of LDL-c with 29% and the degree of triglyceride with 56% [39]. PPAR*δ* was highly expressed in the intestinal tract. In mice with an intestinal epithelial cell-specific deletion of PPAR*δ*, intestinal PPAR*δ* protected against diet-induced obesity, insulin resistance, and dyslipidemia. Furthermore, absence of intestinal PPAR*δ* abolished the ability of PPAR*δ* agonist GW501516 to increase plasma levels of HDL-cholesterol. Besides, the increase in cholesterol transport by inducing the upregulation of ATP-binding cassette transporter A1 (ABCA1) is also related to PPAR*δ* in macrophages, fibroblasts, and intestinal cells [40].

Our study reported that high-fat diet-induced hypertrophy of adipocytes was associated with increased expression of CB1 receptor, which was directly regulated by PPAR*δ* [41]. Obesity is also a main cause of renal disease, which has obvious distinctions from primary glomerulosclerosis because of its special pathologic characteristics. The renal disease associated with obesity includes hypertrophy and glomerulosclerosis with a series of metabolic comorbidities [42]. Our novel finding suggested that glomerular hypertrophy was associated with decreasing PPAR*δ* expression and elevating phosphorylation of p38 MAPK in rats on HFD-induced metabolic syndrome. During exploring the potential drugs for obesity-related hypertension treatment, we found several metabolic regulation effects of telmisartan, kind of angiotensin II receptor blockers (ARBs) being commonly utilized in the therapy of lower blood pressure. Long-term administration of telmisartan significantly reduced visceral fat and prevented high-fat diet-induced obesity in wild-type mice and hypertensive rats but not in PPAR*δ* knockout mice [43].

### 4.2. PPAR*δ* Activation Improves Insulin Resistance and Glucose Homeostasis

Previous studies have demonstrated a potential insulin sensitizing activity of PPAR*δ* agonists. Use of GW501516 could reverse pancreatic islet hypertrophy and increase glucose-stimulated insulin secretion in ob/ob mice [44]. However, subsequent study did not find proinsulin secretion effect of GW501516 in isolated islets [45]. It was proposed that the improvement of glucose metabolism by PPAR*δ* activation was mediated by increasing fatty acid catabolism in muscle. Mice were fed a high fat diet to manifest an improvement in insulin sensitivity in response to activation of PPAR*δ*. Interestingly, this effect was not found in PPAR*δ*-null mice [46]. Similarly, PPAR*δ* activation for three months in db/db mice decreases glucose level in association with improving insulin sensitivity and islet function [47].* In vivo* experiments in mice with upregulated PPAR*δ* expression and activity by adenovirus mediated gene delivery further demonstrated the key role of PPAR*δ* in glucose homeostasis [48]. Meanwhile, Li and her colleagues investigated the effects of telmisartan on insulin signaling and glucose uptake in culturing myotubes and skeletal muscle from wild-type and muscle-specific PPAR*δ* knockout mice [49]. They found that telmisartan treatment could reverse high-fat diet-induced insulin resistance and glucose intolerance in WT but not in muscle-specific PPAR*δ* knockout mice. And the suppressed protein levels of PPAR*δ*, phospho-Akt, phospho-AS160, and Glut4 translocation to the plasma membrane in the skeletal muscle on insulin stimulation were also restored by telmisartan administration.

### 4.3. PPAR*δ* Activation Antagonizes Metabolic Vascular Dysfunction

Atherosclerosis progression is intimately linked with impaired endothelial-dependent diastolic function induced by dyslipidemia and inflammation. Macrophages played an important role in atherosclerosis, which could mediate inflammatory reaction in the control of PPAR*δ*. And the expressions of PPAR*δ* were significantly higher in the process of macrophages differentiation; thus PPAR*δ* has been thought as the target of atherosclerosis therapy [50]. The mechanism of inflammatory macrophages regulated and controlled by PPAR*δ* was special. In fact, the level of inflammatory mediators was lower in gene missing macrophages, and higher in PPAR*δ* overexpressed macrophages. However, PPAR*δ* agonist could reduce the expression of inflammatory gene in macrophages [39]. The further research showed that inflammatory enhancement or anti-inflammatory effect of PPAR*δ* mainly depended on the binding between receptor and ligand binding. With lack of ligand in the environment, PPAR*δ* isolated inflammatory reactions transcribe inhibitor BCL-6, which made BCL-6 become useless and therefore led to inflammatory enhancement. Once activated by the ligand, the BCL-6 was released and presented an anti-inflammatory effect [50]. So PPAR*δ* agonists might become an effective atherosclerosis treatment drug by inhibiting macrophage inflammatory reaction. Besides, the anti-inflammatory effects of PPAR*δ* have been clearly demonstrated* in vivo* and* in vitro*. There were reports that PPAR*δ* agonists significantly enhanced the activities of antioxidant kinase, glutathione peroxidase, and hemeoxygease-1 and developed several defense mechanisms to remove reactive oxygen species (ROS). So PPAR*δ* could prevent endothelial cells from free radicals damage by increasing antioxidant capacity [51, 52]. Toral M et al. also found that chronic activation of PPAR by GW0742 to mice fed with high fat diet (HFD) could prevent the gain of body weight and fat accumulation. Moreover, GW0742 administration increased both aortic Akt and endothelial nitric oxide synthase phosphorylation in the HFD fed mice. Thus, PPAR*δ* might be a potential target for treating obesity-related hypertension [51].

In recent years, the role of PPAR*δ* in the vasculature has attracted much attention. The current studies verified that PPAR*δ* in endothelial cells played a role in the regulation of oxidative injury, inflammation, blood coagulation, cell proliferation, and apoptosis [53, 54]. One study revealed that PPAR*δ* agonists acutely caused vasodilatation, which was partially dependent on endothelial NO synthase (eNOS) activation through the Akt pathway [55]. PPAR*δ* activation in ECs produced an acute transcription-independent regulation of eNOS phosphorylation. Our study showed that the impaired vasorelaxation in MS rats was improved by incubating arteries with rosiglitazone. Importantly, this effect was blocked by inhibition of either PPAR*γ* or PPAR*δ*. In cultured endothelial cells, acute treatment with rosiglitazone increased the phosphorylation of Akt and eNOS and the production of NO. These effects were also abolished by inhibition of PPAR*γ*, PPAR*δ*, or PI3K. In conclusion, rosiglitazone improved endothelial function through both PPAR*γ*- and PPAR*δ*-mediated phosphorylation of Akt and eNOS, which might help to reconsider the complex effects and clinical applications of rosiglitazone [56].

Both sodium and glucose metabolism have been suggested to participate in the pathogenesis of hypertension and increase the risk of cardiovascular events [57, 58]. However, whether there were shared common regulatory mechanisms between sodium and glucose homeostasis was not clear [59]. Our previous study found that PPAR*δ* may regulate both natriuresis and glucose homeostasis through the inhibition of the renal sodium-glucose cotransporter 2 (SGLT2) by adiponectin [60]. Adiponectin downregulated renal SGLT2 expression and function, which in turn reduces the reabsorption of sodium and glucose. However, this mechanism is dampened by hyperglycemia in diabetes. Gordish KL et al. also found that 20% fructose diet could cause salt-sensitive hypertension, leading to the sodium retention when accompanied with high salt diet. As a result, the blood pressure was raised and renal nitric oxide availability was impaired [61]. A most recent study demonstrated that hyperglycemia induced by chronic intraperitoneal and oral glucose loading can increase the expression and activity of Na+-K+-ATPase in renal cortex, which is responsible for increased sodium reabsorption, leading to the increased blood volume and eventual blood pressure [62].

## 5. Conclusion

**Figure 1:**
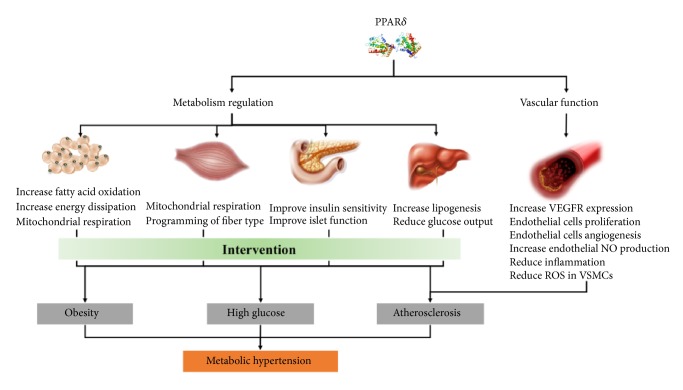
Potential role of PPAR*δ* in the metabolism regulation and vascular function.

Over the past decade, our knowledge of the physiologic function of PPAR*δ* has increased considerably. More evidences suggest that this nuclear receptor plays an important role in the control of metabolic homeostasis and cardiovascular function. Furthermore, through its HDL-C raising, regulation of sodium-glucose homeostasis, anti-inflammatory effect, and protecting vascular endothelial and smooth muscular activities, PPAR*δ* could be a potential target to control atherogenesis and blood pressure (Figure 1). Future experiments combining the use of tissue-specific PPAR*δ* knockout mice and selective PPAR*δ* modulators with better bioactivities shed more light on the therapeutic potential of PPAR*δ* agonists in treating metabolic-related hypertension.
